# Gene Targeting Implicates Cdc42 GTPase in GPVI and Non-GPVI Mediated Platelet Filopodia Formation, Secretion and Aggregation

**DOI:** 10.1371/journal.pone.0022117

**Published:** 2011-07-18

**Authors:** Huzoor Akbar, Xun Shang, Rehana Perveen, Mark Berryman, Kevin Funk, James F. Johnson, Narendra N. Tandon, Yi Zheng

**Affiliations:** 1 Department of Biomedical Sciences, College of Osteopathic Medicine, Ohio University, Athens, Ohio, United States of America; 2 Division of Experimental Hematology and Cancer Biology, Children's Hospital Medical Center, University of Cincinnati, Cincinnati, Ohio, United States of America; 3 Thrombosis Research, Otsuka Maryland Medicinal Laboratories, Rockville, Maryland, United States of America; University of Bergen, Norway

## Abstract

**Background:**

Cdc42 and Rac1, members of the Rho family of small GTPases, play critical roles in actin cytoskeleton regulation. We have shown previously that Rac1 is involved in regulation of platelet secretion and aggregation. However, the role of Cdc42 in platelet activation remains controversial. This study was undertaken to better understand the role of Cdc42 in platelet activation.

**Methodology/Principal Findings:**

We utilized the Mx-cre;Cdc42^lox/lox^ inducible mice with transient Cdc42 deletion to investigate the involvement of Cdc42 in platelet function. The Cdc42-deficient mice exhibited a significantly reduced platelet count than the matching Cdc42^+/+^ mice. Platelets isolated from Cdc42^−/−^, as compared to Cdc42^+/+^, mice exhibited (a) diminished phosphorylation of PAK1/2, an effector molecule of Cdc42, (b) inhibition of filopodia formation on immobilized CRP or fibrinogen, (c) inhibition of CRP- or thrombin-induced secretion of ATP and release of P-selectin, (d) inhibition of CRP, collagen or thrombin induced platelet aggregation, and (e) minimal phosphorylation of Akt upon stimulation with CRP or thrombin. The bleeding times were significantly prolonged in Cdc42^−/−^ mice compared with Cdc42^+/+^ mice.

**Conclusion/Significance:**

Our data demonstrate that Cdc42 is required for platelet filopodia formation, secretion and aggregation and therefore plays a critical role in platelet mediated hemostasis and thrombosis.

## Introduction

Cdc42, a member of the Rho family of small GTPases, has been implicated in regulation of numerous cellular processes including organization of actin cytoskeleton, cell-cell and cell-extracellular matrix adhesion, and spreading [1]–[6]. Although Cdc42 was reported to be present in platelets more than twenty-two years ago [7], a better understanding of its role in regulation of platelet activation has remained elusive. Binding of agonists such as thrombin-receptor activated peptide (TRAP) to its receptors on platelets has been shown to induce integrin α_IIb_β_3_ mediated translocation of Cdc42 to the cytoskeleton in a reversible manner [8]. Out-side in signaling in platelets initiated by binding of von Willebrand factor (vWf) to platelet GP Ib-IX has been reported to induce activation of Cdc42 that involves interaction of GP 1b-IX with 14-3-3ζ [9]. Moreover, it has been shown that thrombin induced activation of platelets results in accumulation of GTP-bound active form of Cdc42 [10] and that Cdc42 dependent activation of p21-activated kinase (PAK) induces platelet lamellipodia formation [11].

To date, the role of Cdc42 in platelet filopodia formation, secretion and aggregation remains unclear. One report has suggested that pharmacologic inhibition of Cdc42 inhibits platelet adhesion to immobilized collagen as well as aggregation induced by collagen but does not inhibit adhesion of platelets to CRP or fibrinogen or platelet aggregation induced by CRP or thrombin [12]. Another report has shown that constitutive megakaryocyte/platelet-specific deletion of Cdc42 in mice results in increased secretion and aggregation responses to diverse agonists as well as increased platelet aggregate formation on collagen under flow [13]. The contradictory data and conclusions presented in these two reports fail to clarify the role of Cdc42 in platelet activation. To more clearly define the role of Cdc42 in platelet activation we have utilized Cdc42 null platelets from an inducible Cdc42 knockout mouse model obtained by a different gene targeting strategy [14]. Mouse embryonic fibroblasts and T-cells, as well as other lineages, from this mouse strain have been shown to be readily rescued in various phenotypes from filopodia induction, directional migration, proliferation, and differentiation to survival by reconstitution with WT Cdc42 [15], [16]. Here we report that the mice with inducible deletion of Cdc42 in blood cells exhibit not only thrombocytopenia and prolonged bleeding times but also display severe defects in filopodia formation and aggregation possibly due to inhibition of secretion and blockade of Akt phosphorylation regardless of the nature of agonists employed. These consistent phenotypes and mechanisms establish a bona fide physiological role of Cdc42 in the regulation of platelet activation induced by agonists that activate platelets by GPVI-dependent as well as GPVI-independent signaling.

## Materials and Methods

### Materials

Chemicals and reagents were purchased either from Sigma-Aldrich or from specifically noted sources. Collagen was obtained from Chrono-Log Corporation. Anti-Cdc42 antibody was purchased from BD Biosciences and anti-PAK and p-PAK1/2, Akt and p-Akt antibodies were obtained from Cell Signaling. Collagen related peptide (CRP) was synthesized as reported earlier [17].

### Methods

#### Mouse maintenance, blood collection and preparation of washed mice platelet suspensions

All experiments using mice were performed according to the protocols approved by the Institutional Animal Care and Use Committees at the Children's Hospital Research Foundation (IACUC Protocol#8D06052), Cincinnati, Ohio and at Ohio University (IACUC Protocol#H08-12) Athens, Ohio. The Mx-cre, Mx-cre;Cdc42^loxP/loxP^ mice were generated previously [14], [18]. Inducible deletion of Cdc42 GTPase from the platelets of the mice was achieved by 4–5 intra-peritoneal injections of poly (I∶C) as described [14]. Platelets from the Mx-cre;Cdc42^WT/WT^ mice given 4–5 intra-peritoneal injections of poly (I∶C) were used as control samples. Protocol for drawing blood and washing murine platelets is essentially the same as we described earlier [19]. Blood was drawn by cardiac puncture from anesthetized wild type (WT) and Cdc42 deficient mice into a syringe containing 160 µl of ACD (2.5% trisodium citrate, 2% dextrose, 1.5% citric acid) and transferred into tubes containing 500 µl of HEPES-buffered Tyrode's solution. Platelets were isolated by centrifugation at 90g for 10 minutes, immediately after adding 5 mM EDTA [19]–[21]. The remainder of blood was diluted to 1.5 ml with HEPES-buffered Tyrode's solution, pH 6.5 and centrifuged at 90g for 10 minutes to recover additional platelets. Platelets were washed three times, in the presence of apyrase (0.4 U/ml), an enzyme that hydrolyzes ADP, and 2.0% EGTA, with HEPES-buffered Tyrode's solution, pH 6.5 and finally resuspended in HEPES-buffered Tyrode's solution without calcium, pH 7.4 and counted in Hemavet 950FS (Drew Scientific, CT, USA). The platelet count was adjusted to 2.5×10^8^ per ml for aggregation studies.

#### Platelet actin structures on fibrinogen and collagen-related peptide

Glass cover slips were coated with fibrinogen or collagen-related peptide overnight at 4°C. Non-specific binding was blocked by incubating cover slips with bovine serum albumin (BSA, 1%) in phosphate-buffered saline (PBS) at 37°C. Cover slips were rinsed with Tyrode's-HEPES buffer after removing BSA. Washed platelets from Cdc42^+/+^ and Cdc42^−/−^ mice were layered over cover slips and incubated at 37°C for time periods shown in figure legends. The cover slips were rinsed with PBS to remove free platelets. Platelets on cover slips were then fixed with 4% paraformaldehyde for ten minutes, rinsed with PBS twice and permeabilized with 0.1% Triton X-100 for 60 seconds. After two rinses with PBS platelets were stained with Alexa 594-phalloidin to visualize F-actin. Matching immuno-fluorescence and differential interference contrast (DIC) images were taken with a Nikon E-600 microscope using a Plan Apo 60x/1.4 oil objective. Digital photos were recorded with a Spot camera using Spot Advanced software version 4.7 and Photoshop was used to auto adjust brightness and contrast. Platelet spreading was quantified using the Image J software (http://rsbweb.nih.gov/ij).

#### Assessment of P-selectin expression, secretion of ATP and platelet aggregation

P-selectin release was assessed as described earlier [19]. The PRP was incubated with 1 mM aspirin at 37°C for 30 minutes and then platelets were isolated by centrifugation, washed twice and finally resuspended in HEPES-buffered Tyrode's solution without calcium, pH 7.4 containing 0.2% bovine serum albumin and apyrase (0.1 U/ml). Washed platelets (1–1.5×10^6^) were incubated with 10 µl of FITC-conjugated anti-CD62P (P-selectin) antibody solution for 30 minutes at 37°C without stirring. Expression of P-selectin on platelet surface was quantified by flow cytometry (FACSCalibur, Becton-Dickinson) and the Cellquest software program [22]. Secretion of ATP from the dense granules was assessed by a luminescence method using a luciferin/luciferase kit from Chrono-Log Corporation (Havertown, PA) [19]. The luciferin/luciferase reagent was added to platelets one minute prior to addition of CRP. Platelet aggregation was monitored as reported earlier [19], [20] using a Lumi-Aggregometer at 37°C and a stirring speed of 900 rpm. Washed Cdc42^+/+^ and Cdc42^−/−^ platelets were stimulated with CRP, collagen or thrombin and aggregation was recorded for 4 minutes.

#### Phosphorylation of PAK1/2 and Akt

Washed murine platelets were stimulated with thrombin or CRP for a specified time period. The reactions were terminated by addition of 5× sample buffer. Western blotting of PAK1/2, p-PAK1/2, Akt, and p-Akt, and β-actin were done as reported earlier [14].

#### Tail bleeding time measurement

Tail bleeding time measurements were performed as described earlier [19]. Mice were anesthetized with and kept under a constant flow of 2.5% isoflurane and 0.35% of oxygen. The tip of the tail at 1.5 mm diameter was cut and immediately immersed in saline at 37°C. The bleeding time was defined as the time needed for the cessation of visible blood stream for one minute [23]. Monitoring of the bleeding times was stopped at 15 minutes by cauterizing the tails to prevent excessive loss of blood.

#### Statistical analysis

Data are expressed as means ± SD or SEM (as described in figure legends). Statistical significance between the bleeding times of the Cdc42^+/+^ and Cdc42^−/−^ mice was assessed by student's *t*-test. The *p* values of less than 0.05 are considered statistically significant.

## Results

### Inducible deletion of Cdc42 from bone marrow results in thrombocytopenia

**Figure 1:**
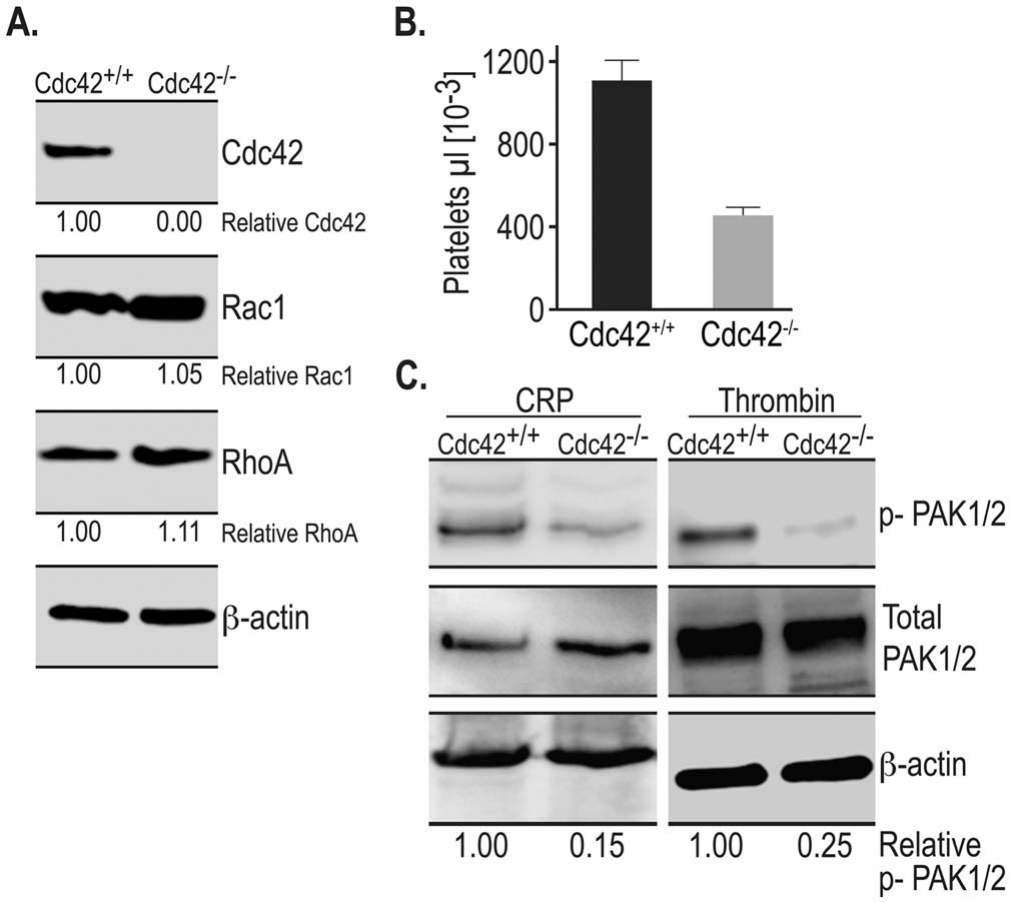
Gene targeting of Cdc42 induced thrombocytopenia and blocked signaling downstream of Cdc42. A, Expression of Cdc42, Rac1 and RhoA in platelet lysates from the wild type and genetically targeted mice was probed by Western blotting of Cdc42, Rac1 and RhoA. Platelets from Cdc42 gene targeted mice as compared to the poly (I∶C) treated matching wild type mice showed a complete lack of Cdc42 GTPase. The expression of Rac1 and RhoA was not altered in Cdc42^−/−^ platelets. β-actin expression was used as a loading control. B, Platelet counts (Mean ± SEM) in the Cdc42^−/−^ mice (n = 11) were significantly lower (*p*<0.05) than the platelet counts in the Cdc42^+/+^ mice (n = 12). C, CRP (0.2 µg/ml) or thrombin (0.1 U/ml) induced phosphorylation of PAK1/2 is inhibited in the Cdc42^−/−^ mice platelets as compared to the platelets from Cdc42^+/+^ mice. Phosphorylation of PAK1/2 was analyzed as described in the methods section.

An interferon-inducible method was used to generate conditional knockout mice lacking Cdc42 in hematopoietic cells including platelets, in the Mx-cre:Cdc42 ^loxP/LoxP^ mice [14]. Loss of Cdc42 in the platelets isolated from the Mx-cre:Cdc42 ^loxP/LoxP^ mice 5–10 days after the poly(I∶C) treatment was assessed by Western blotting. As shown in Fig. 1A, Cdc42 GTPase is expressed in platelets from the Cdc42^+/+^ but not in platelets from Cdc42^−/−^ mice. We investigated the possibility that inducible deletion of Cdc42 may have affected expression of Rac1 or RhoA. Blots in Fig. 1A demonstrate that expression of neither Rac1 nor RhoA is altered by deletion of Cdc42. The platelet count in Cdc42^−/−^ mice was significantly lower than that in the Cdc42^+/+^ mice (Fig. 1B). Platelet volume (Means ± SD) was increased in Cdc42^−/−^ mice (5.45±0.22 fl, n = 11) as compared to Cdc42^+/+^ mice (4.92±0.24 fl, n = 12). Our findings of thrombocytopenia and an increase in platelet volume in Cdc42-deficient mice are in agreement with a recent report of thrombocytopenia and an increase in the size of platelets in mice constitutively lacking Cdc42 in megakaryocytes and platelets [13], and confirm that Cdc42 is involved in platelet production.

### Activation of PAK1/2 is significantly diminished in Cd42^−/−^ platelets

Platelet agonists such as thrombin, TRAP and collagen all have been shown to induce activation of Rac1 as well as Cdc42 [10], [11], [19]. Activation of Rac1 or Cdc42 GTPase leads to phosphorylation of PAK1/2, the putative effector molecule, in platelets and other cell types [11], [24], [25]. We have shown previously that genetic or pharmacologic targeting of Rac1 prevents phosphorylation of PAK1/2 [19]. To examine if PAK1/2 activity is also regulated by Cdc42 in platelets, washed platelets from the Cdc42^+/+^ and Cdc42^−/−^ mice were stimulated with collagen-related peptide (CRP) or thrombin for two minutes and phosphorylation of PAK1/2 was assessed by Western blotting. As shown in Fig. 1C, CRP- or thrombin-induced phosphorylation of PAK1/2 in Cdc42^−/−^, as compared to Cdc42^+/+^, platelets is diminished by 85% and 75% respectively. These findings that inducible deletion of Cdc42 prevents agonist induced signaling downstream of Cdc42 further validate the use of this Cdc42-deficient mouse model for investigating the role of Cdc42 in platelet activation.

### Cdc42^−/−^ platelets fail to form filopodia or lamellipodia on fibrinogen or CRP

**Figure 2:**
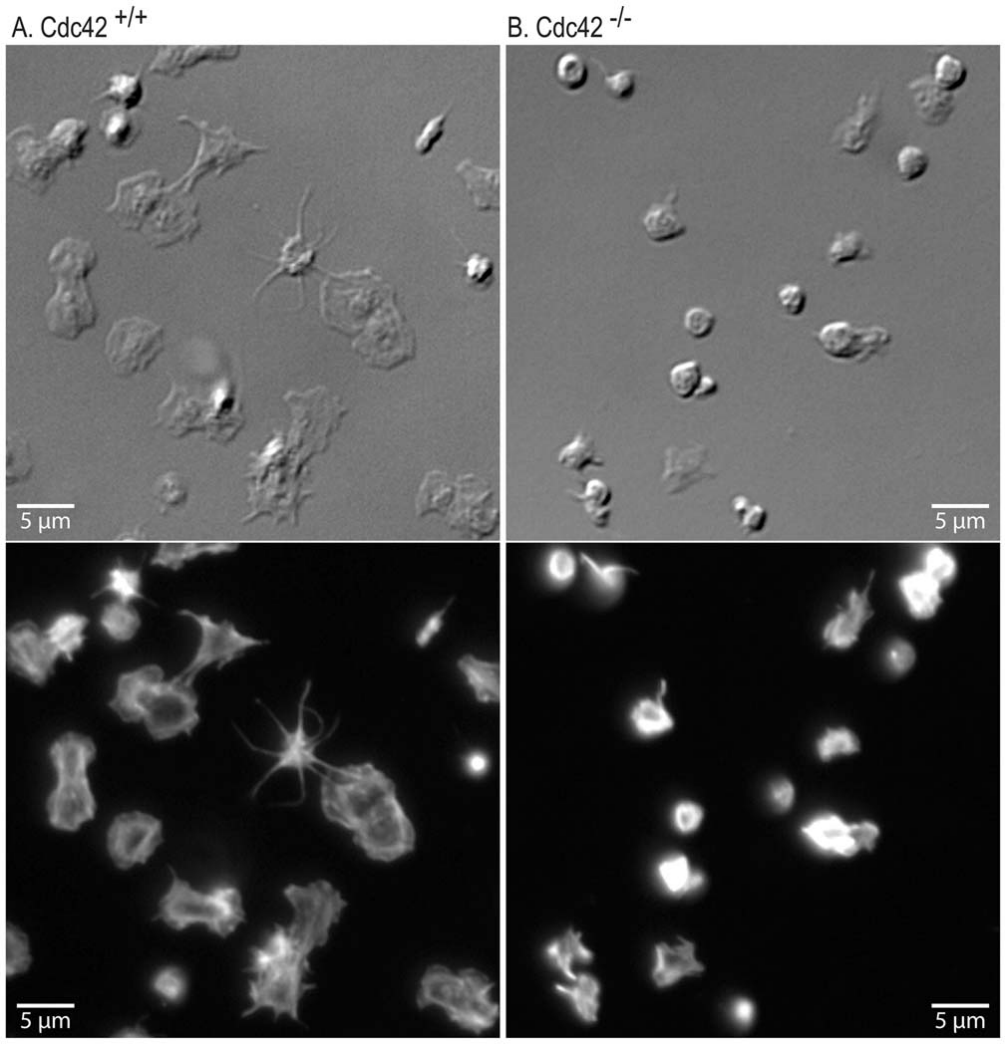
Inducible genetic targeting of Cdc42 inhibited filopodia and lamellipodia formation on immobilized collagen-related peptide. Washed platelets from Cdc42^+/+^ (A) and Cdc42^−/−^ (B) mice were layered over cover slips coated with CRP (0.5 µg/ml) for 20 minutes. The cover slips were washed and adherent platelets were processed for DIC (top panel) and immuno-fluorescence (bottom panel) microscopy as detailed in the methods section. Platelets from Cdc42^−/−^ mice failed to form filopodia or lamellipodia on CRP.

**Figure 3:**
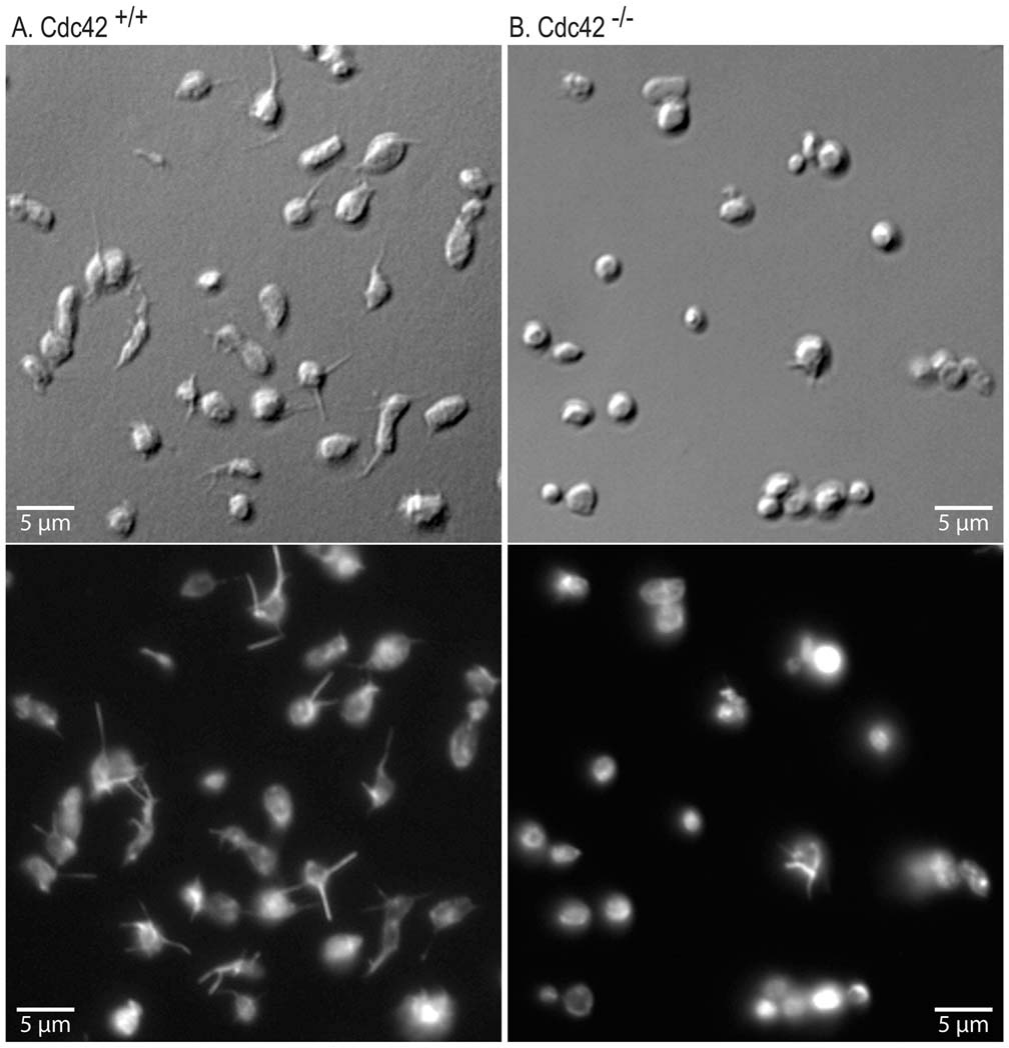
Inducible genetic targeting of Cdc42 inhibited filopodia formation on immobilized fibrinogen. Washed platelets from Cdc42^+/+^ (A) and Cdc42^−/−^ (B) mice were layered over cover slips coated with fibrinogen (3.0 µg/ml) for 20 minutes. The DIC (top panel) and immuno-fluorescence (bottom panel) images show that platelets from Cdc42^−/−^ mice failed to form filopodia on immobilized fibrinogen.

**Figure 4:**
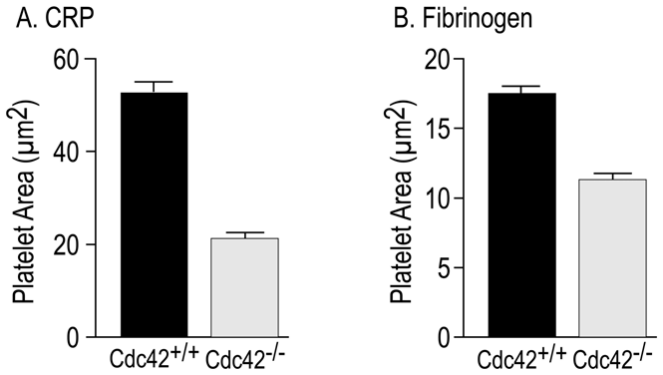
Inducible genetic targeting of Cdc42 inhibited platelet spreading on immobilized CRP and fibrinogen. Spreading of washed platelets from Cdc42^+/+^ and Cdc42^−/−^ mice on CRP or fibrinogen was quantified using Image J software (http://rsbweb.nih.gov/ij). Each bar represents mean surface are ± SEM of 100 platelets. The spreading of Cdc42^−/−^ platelets on immobilized CRP or fibrinogen was significantly (p<0.01) diminished as compared to the Cdc42^+/+^ platelets.

Cdc42 is known to regulate actin polymerization and filopodia formation, as well as spreading, on cellular and extra-cellular matrices in several cell types [26], [27]. We investigated the effect of deficiency of Cdc42 on platelet actin structures on immobilized fibrinogen or CRP to determine the role of Cdc42 in integrin α_IIb_β_3_ and GPVI mediated platelet morphological changes respectively. Aspirin treated washed Cdc42^+/+^ and Cdc42^−/−^ mice platelets were layered over immobilized fibrinogen or CRP in the presence of apyrase and morphological changes were assessed by immuno-fluorescence and DIC microscopy. Platelets from Cdc42^−/−^ mice, as compared to Cdc42^+/+^ mice, failed to form filopodia or lamellipodia on immobilized CRP (Fig. 2). The micrographs in Fig. 3 show that Cdc42^−/−^ platelets failed to form filopodia on immobilized fibrinogen. The spreading of Cdc42^−/−^ platelets on immobilized CRP (21.3±1.3 µm^2^) or fibrinogen (11.3±0.4 µm^2^) was significantly diminished as compared to the Cdc42^+/+^ platelets spreading on CRP (52.7±1.9 µm^2^) or fibrinogen (17.5±0.5 µm^2^) respectively (Fig. 4).

The inability of Cdc42^−/−^ platelets to form filopodia or lamellipodia either on fibrinogen or CRP suggests that Cdc42 plays an important role not only in integrin α_IIb_β_3_ mediated out-side in signaling but also GPVI mediated inside-out signaling that promotes binding of collagen to integrin α_2_β_1._ Failure of Cdc42^−/−^ platelets to form filopodia on CRP, a GPVI specific agonist, further supports a critical role for Cdc42 in GPVI mediated signaling in platelet activation.

### Gene targeting of Cdc42 inhibits secretion from the alpha and the dense granules

**Figure 5:**
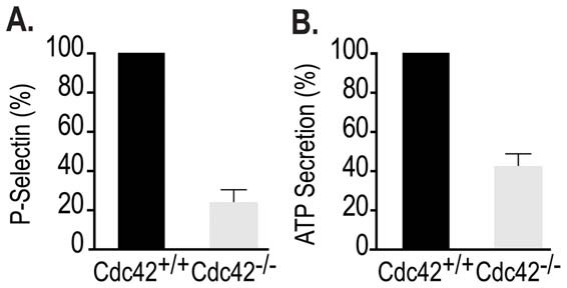
Genetic targeting of Cdc42 inhibited release of p-selectin from α-granules and secretion of ATP from dense granules. A, Thrombin (0.2 U/ml) induced expression of P-selectin and B, CRP (0.2 µg/ml) induced secretion of ATP is inhibited in platelets from Cdc42^−/−^ mice compared with the platelets from Cdc42^+/+^ mice. Bar graphs are the means ± SD (n = 3).

Rac1 and Cdc42 have been implicated in regulating exocytosis [28]–[33]. Cdc42 has been shown to be involved in second phase of insulin granule exocytosis in pancreatic islet beta cells [29] as well as glucagon-like peptide-1 secretion in the intestinal endocrine L cell [34]. We have observed that Rac1 is involved in secretion from platelet granules [19]. We investigated the role of Cdc42 in secretion by quantifying release of P-selectin from the α-granules and secretion of ATP from the dense granules in platelets from Cdc42^+/+^ and Cdc42^−/−^ mice. The data in Fig. 5 show that thrombin or CRP induced release of P-selectin and secretion of ATP in Cdc42^−/−^ platelets, as compared to Cdc42^+/+^ platelets, is diminished. These findings implicate Cdc42 as a positive regulator of platelet secretion.

### Platelet aggregation induced by thrombin, CRP or collagen is inhibited in Cdc42^−/−^ platelets

**Figure 6:**
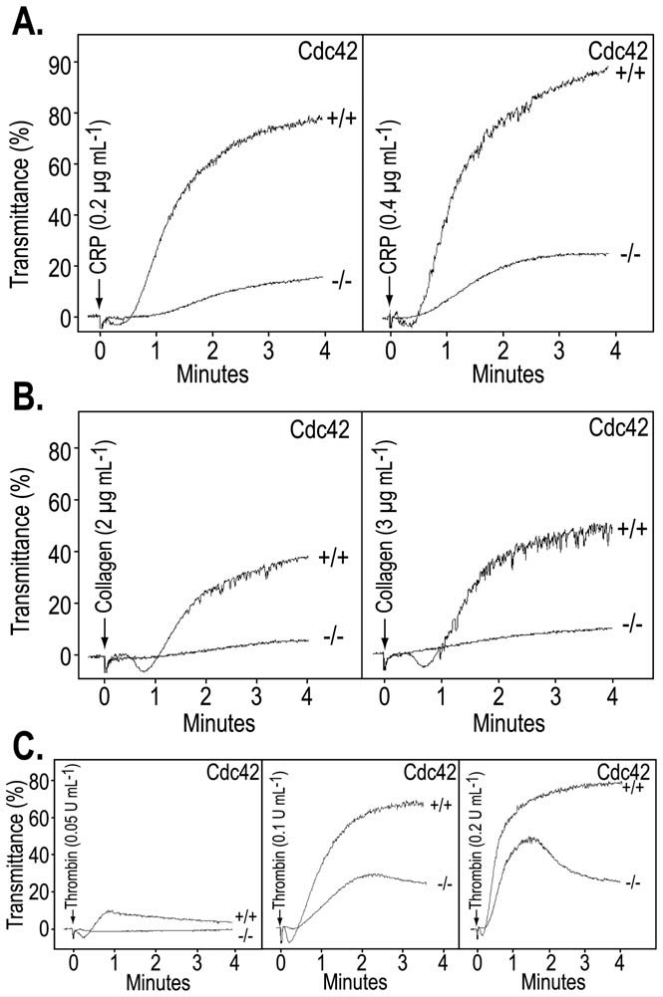
Deficiency of Cdc42 inhibited platelet aggregation induced by collagen, CRP or thrombin. Washed platelets from Cdc42^+/+^ and Cdc42^−/−^ were stimulated by addition of CRP (A), collagen (B) or thrombin (C) and aggregation was recorded using a Lumi-Aggregometer at 37°C and a stirring speed of 900 rpm. Platelets from Cdc42^−/−^ mice compared with the platelets from the Cdc42^+/+^ mice exhibited diminished aggregation induced by all three agonists. The aggregation tracings are representative of three experiments.

In platelets, agonist-receptor mediated inside-out signaling induces exposure of integrin α_IIb_β_3_. Binding of fibrinogen to α_IIb_β_3_ culminates in platelet aggregation. Agonist induced secretion, particularly ADP from the dense granules, plays a pivotal role in aggregation induced by diverse agonists [35]. We investigated the possibility that deficiency of Cdc42 may also inhibit platelet aggregation in part due to diminished secretion. Threshold concentration of thrombin (0.05 U/ml) induced nominal aggregation in Cdc42^+/+^ platelets without inducing any discernable aggregation response in Cdc42^−/−^ platelets (Fig. 6). Increasing concentrations of thrombin (0.1–0.2 U/ml) induced maximal aggregation in Cdc42^+/+^ platelets. Aggregation responses at all thrombin concentrations were diminished in Cdc42^−/−^ platelets (Fig. 6). It is important to note that in Cdc42^−/−^ platelets only primary or reversible aggregation response was observed with increasing concentrations of thrombin. The lack of secondary aggregation response in Cdc42^−/−^ platelets that depends on secretion of ADP suggests that inhibition of secretion due to Cdc42-deficiency is at least partially responsible for the diminished aggregation.

Addition of CRP (0.2–0.4 µg/ml), a GPVI specific agonist, to Cdc42^+/+^ platelets induced a typical aggregation responses but only induced nominal aggregation response in Cdc42^−/−^ platelets (Fig. 6). Similarly addition of collagen (2–3 µg/ml) to Cdc42^+/+^ platelets induced a typical aggregation pattern. In contrast, platelets from Cdc42^−/−^ platelets failed to undergo aggregation upon stimulation with collagen (Fig. 6). The diminished platelet aggregation responses to thrombin, CRP or collagen seen in Cdc42^−/−^ platelets indicate that Cdc42 plays an important role in aggregation induced by GPVI- and non-GPVI mediated platelet activation.

### Agonist-induced activation of Akt is diminished in Cdc42-deficient platelets

**Figure 7:**
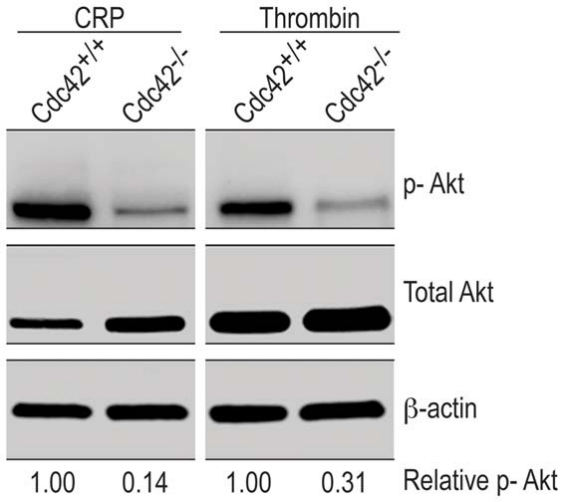
Agonist-induced phosphorylation of Akt is reduced in Cdc42^−/−^ platelets. Washed platelets from Cdc42^+/+^ and Cdc42^−/−^ mice were incubated with CRP (0.2 µg/ml) or thrombin (0.1 U/ml) for 5 minutes at 37°C with constant stirring in a Chrono-Log aggregometer. The reactions were terminated by adding 5× sample buffer, processed for Western blotting and probed for Akt, p-Akt and β-actin as detailed in methods.

Diverse platelet agonists induce phosphorylation of Akt [36]–[41]. Phosphorylation of Akt has been linked to secondary aggregation [42]. Secreted ADP induces Akt phosphorylation via Gα_i_ mediated signaling [39]. However, phosphorylation of Akt induced by convulxin, a GPVI specific agonist, is mediated, in part, by secreted ADP and in part by activation of PI3Kβ [43]. We investigated the role of Cdc42 in thrombin and CRP induced activation of Akt. The data in Fig. 7 show that CRP or thrombin induced phosphorylation of Akt is inhibited in Cdc42^−/−^ platelets. These findings suggest that Cdc42 is critical in Akt phosphorylation mediated by diverse signaling mechanisms and consequent platelet aggregation.

### Cdc42-deficient mice exhibit prolonged tail bleeding times

**Figure 8:**
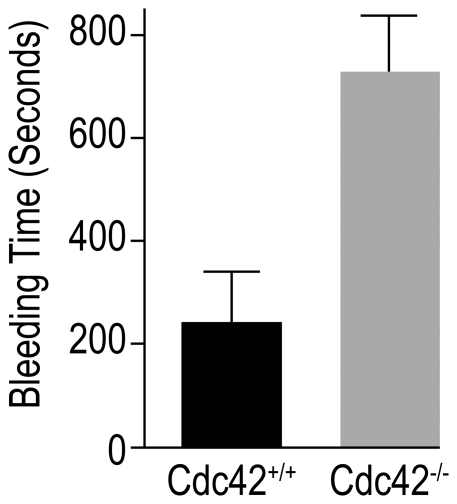
Deficiency of Cdc42 prolonged tail bleeding time. The tail bleeding times were assessed in Cdc42^+/+^ (n = 8) and Cdc42^−/−^ (n = 8) mice as detailed in methods. Bar graphs are the means ± SEM of the bleeding times. Cdc42-deficient, as compared to Cdc42^+/+^, mice exhibited significantly prolonged (*p*<0.05) bleeding times.

Platelet aggregation at the site of injury constitutes the primary hemostatic response that ultimately leads to arrest of bleeding. A defective or diminished aggregation may slow or prevent hemostasis. We hypothesized that diminished aggregation in Cdc42-deficient mice may lead to a delayed hemostasis. To test this possibility we performed tail bleeding time assay in Cdc42^+/+^ and Cdc42^−/−^ mice as described earlier [19]. The data in Fig. 8 show that Cdc42^−/−^ mice, as compared to Cdc42^+/+^ mice, had a significantly prolonged bleeding time. The prolonged tail bleeding times taken together with diminished aggregation responses in Cdc42^−/−^ mice strongly suggest that Cdc42 regulates platelet dependent primary hemostasis.

## Discussion

Cdc42 is a member of the Rho family of small GTPases and has been known to regulate actin organization and associated morphological changes such as filopodia formation in a variety of cell types. However, its role in platelet activation, particularly filopodia formation, granular secretion and aggregation, is controversial at this time due to recent contradictory reports [12], [13]. This study was undertaken to more clearly define the role of Cdc42 in regulation of platelet activation using an inducible Cdc42 knockout mouse model. The data in Fig. 1A show that gene targeting of Cdc42 resulted in a lack of expression of Cdc42 but did not affect expression of related Rac1 and RhoA. Our findings that inducible gene targeting of Cdc42 results in a significant thrombocytopenia (Fig. 1) and prolonged tail bleeding times in Cdc42-deficient mice (Fig. 8) demonstrate that Cdc42 is essential for normal platelet function. These observations are in agreement with a recent report that megakaryocyte/platelet specific constitutive deletion of Cdc42 results in thrombocytopenia as well as prolonged tail bleeding times [13].

We have previously reported that deletion of Cdc42 leads to hematopoietic stem/progenitor cell defects in differentiation, cell cycle progression, and homing/lodging in the bone marrow [14], [44]. Thus, the observed partial reduction in platelet production in Cdc42 deficient mice may be due to early progenitor defects as well as defects in megakaryocyte differentiation. The mechanisms by which Cdc42 regulates platelet production remain unclear at this time. The interferon inducible deletion of Cdc42 in Mx-cre;Cdc42^lox/lox^ mice affects all blood lineages including the stem/early progenitor compartments and therefore makes this particular mouse model unsuitable for studying the developmental mechanism of a terminal lineage like platelet.

Wiskott-Aldridge Syndrome (WAS) patients exhibit thrombocytopenia [45]. Since Wiskott-Aldridge Syndrome Protein (WASP) is regulated by Cdc42, deficiency of Cdc42 may be involved in thrombocytopenia. However, Cdc42 engages multiple effector pathways including PAK, WASP, IQGAP, Par6 [16], making it difficult to sort out the specific contribution of WASP to the diminished platelet number in Cdc42-deficient mice. Technical difficulties in reintroducing Cdc42 mutants (e.g. defective in WASP binding) to platelets pose another challenge for demonstrating a causal relationship between Cdc42 deficiency, defective WASP activation and thrombocytopenia. More recently WASP has been shown to play an important role in regulation of integrin α_IIb_β_3_ mediated platelet activation [45] and it is possible that this pathway is involved in Cdc42 mediated α_IIb_β_3_ regulation.

The p-21activated kinases (Paks) are known effector molecules of Cdc42 and have been linked with actin polymerization and lamellipodia formation. The Paks have been shown to be phosphorylated in platelets by thrombin, TRAP as well as convulxin, a GPVI agonist [11], [46]. Our finding that CRP, a GPVI specific agonist, as well as thrombin induced phosphorylation of PAK1/2 is inhibited in Cdc42-deficient platelets (Fig. 1) confirms not only deletion of Cdc42 in the gene targeted mice but also demonstrates that signaling downstream of Cdc42 has been effectively blocked in Cdc42^−/−^ platelets. This is a first report to show that the Cdc42 is involved in GPVI-specific signaling leading to phosphorylation of PAK1/2 in murine platelets. We have reported earlier that gene targeting or pharmacologic inhibition of Rac1 also inhibits activation of PAK1/2 [19]. Thus, PAK1/2 appears to be subjected to a dual regulation by Cdc42 and Rac1 in platelets. This suggests that Cdc42/Rac1 either function sequentially or in parallel on this “shared” effector. How PAK1/2 is regulated by both Cdc42 and Rac1 in platelets is not clear at this point. One possibility is that the regulation of PAK1/2 by Cdc42/Rac1 may involve heterodimerization of PAK1/2 and binding of the PAK1/2 dimer to Cdc42/Rac1 simultaneously. Alternatively, Cdc42 and Rac1 may act sequentially in a signaling cascade leading to PAK regulation. Investigations of these hypotheses to resolve the functional relationship between Cdc42 and Rac1 would require multiple gene targeting and transgenic approaches as most standard gene transfer techniques are not applicable in platelets.

Agonists that induce platelet activation also activate Cdc42 [10] and active Cdc42 can initiate platelet actin cytoskeleton reorganization [9] leading to lamellipodia [11] and filopodia formation [47]. Ligand density has been shown to affect integrin α_IIb_β_3_-mediated platelet signaling and spreading [48] and increasing agonist concentrations, at least partially, reverse the diminished aggregation responses observed in Cdc42 deficient platelets (Fig. 6). We therefore utilized lower concentrations of immobilized fibrinogen (3 µg/ml) and CRP ((0.5 µg/ml) to investigate the role of Cdc42 in platelet morphological changes. Our findings that Cdc42^−/−^ platelets failed to form filopodia or lamellipodia and exhibited diminished spreading on immobilized fibrinogen or CRP (Figs. 2, 3, and 4) clearly demonstrate that Cdc42 is involved in reorganization of actin cytoskeleton. The inability of Cdc42^−/−^ platelets to form filopodia on immobilized fibrinogen clearly demonstrate that Cdc42 is essential for integrin α_IIb_β_3_-depedent outside-in signaling involved in reorganization of actin cytoskeleton. The findings that murine platelets were able to form filopodia on immobilized CRP show that GPVI is involved in filopodia formation. Moreover, our observations that Cdc42^−/−^ platelets were unable to form filopodia on CRP clearly demonstrate that Cdc42 is critical for the GPVI mediated actin reorganization. Using secramine A, a non-specific inhibitor, Pula and Poole [12] have suggested that inhibition of Cdc42 partially blocks spreading of platelets on collagen, CRP and fibrinogen. However, our findings disagree with a recent report showing that megakaryocytes/platelets specific deletion of Cdc42 had no effect on filopodia formation on immobilized fibrinogen or CRP [13]. The reason for this discrepancy is not clear at this time. These investigators have also suggested that Cdc42-deficient platelets exhibit specific defect in GPIb-specific signal as evidenced by the reduced extension of filopodia on immobilized vWf. However, they used a significantly lower concentration of vWf (10 µg/ml) as compared to the ten-fold excessive amounts of fibrinogen or CRP. It is possible that platelets from their Cdc42^−/−^ mice would have exhibited defective filopodia formation if significantly lower concentrations of fibrinogen or CRP were used. Another possible explanation for the discrepancy may be that constitutive, but not transient inducible deletion of Cdc42, in megakaryocytes/platelets results in compensatory expression and/or activation of Cdc42-related Rho GTPases such as TC10, TCL or Rif. Such differences between the two knockout systems are difficult to confirm at this time due to the lack of specific antibodies and probes.

Cdc42 has been shown to be critical in exocytosis in a number of cell types [29], [34]. Our findings that platelets from mice with inducible deletion of Cdc42 exhibit diminished secretion from platelet granules (Fig. 5) suggest that Cdc42 plays a critical role in platelet secretory responses. However, Pleines *et al.* [13] have shown that platelets from mice with constitutive deletion of Cdc42 exhibit increased secretory responses. There is no apparent mechanistic explanation for the decreased secretion observed by us (Fig. 5) and the enhanced secretion reported by them. We quantified secretion in washed murine platelets using a luciferin/luciferase chemi-luminescence method. They quantified ATP in supernatants of PRP from heparinized blood after terminating the reactions with EDTA and fixing platelets with formaldehyde. The differences in methodology for assessing secretion, in addition to above discussed genetic models, may account for the differences in these observations.

The functional consequence of platelet activation reactions is aggregation or bridging of platelets that occurs due to binding of fibrinogen to integrin α_IIb_β_3_ on the adjoining platelets. Diverse agonists bind to their specific receptors on platelets and initiate signaling cascades leading to the so-called inside-out signaling that culminates in secretion from platelet granules and a many-fold increase in exposure of integrin α_IIb_β_3_ on platelet surface and consequently a much greater aggregation response. If secretion from platelet granules is essential for maximal, or the so-called irreversible, platelet aggregation response then diminished secretion responses observed in Cdc42^−/−^ platelets (Fig. 5) would result in diminished platelet aggregation response. Indeed our findings that platelet aggregation responses induced by thrombin, collagen or CRP are either minimal or diminished in Cdc42^−/−^ platelets (Fig. 6) suggest that diminished secretion, at least in part, is responsible for impaired aggregation responses in Cdc42^−/−^ platelets. Impaired aggregation responses induced by diverse agonists such as thrombin, collagen or CRP imply that a common rather than an agonist-specific signaling mechanism is responsible for defective platelet aggregation in Cdc42^−/−^ mice. Agonist induced activation of Akt is one such common signaling mechanism. Secondary or irreversible platelet aggregation has been linked to phosphorylation of Akt [42]. Secreted ADP, regardless of the nature of the agonist employed, induces phosphorylation of Akt via activation of Gα_i_ [39] and GPVI specific agonists such as convulxin induce phosphorylation of Akt in part due to secreted ADP and in part due to activation of PI3Kβ [43]. Our findings that CRP, a GPVI specific agonist, and thrombin, that activates murine platelets via PAR3/4 receptors, both induced minimal or only reversible aggregation (Fig. 6) as well as induced only nominal phosphorylation of Akt in Cdc42^−/−^, as compared to the Cd42^+/+^, platelets (Fig. 7) suggest that deficiency of Cdc42 results in defective platelet aggregation, at least in part, due to inhibition of phosphorylation of Akt.

Our observation that Cdc42^−/−^ platelets exhibit impaired platelet aggregation in response to CRP, collagen or thrombin differ from the report showing that platelets from constitutively deleted Cdc42 mice exhibit increased aggregation responses upon stimulation with collagen or CRP and a similar aggregation response upon stimulation with thrombin [13]. These investigators have suggested that increased aggregation induced by collagen and CRP is due to enhanced secretion from platelet granules. There is no obvious explanation for the different observations made by them and us. Again, methodological differences may be attributed to the different findings. However, our data (Fig. 8) and their observation of a prolonged bleeding time in Cdc42^−/−^ mice are hard to reconcile with enhanced secretion/aggregation responses in Cdc42^−/−^ platelets.

Pula and Poole [12] have reported that secramine A blocks collagen but not CRP induced platelet aggregation. However, the non-selective nature of the inhibitor as well as the excessively high concentration of collagen (30 µg/ml) and CRP (5 µg/ml) used in this study make it difficult to compare their data with our data in Cdc42^−/−^ platelets where we used 10-times lower concentration of collagen and CRP. It is possible that secramine A might have inhibited normal i.e. typical aggregation response induced by a lower concentration of CRP.

Platelet aggregation at the site of injury forms the primary hemostatic plug leading to an arrest of bleeding. Standardized bleeding times are considered a measurement of platelets functional integrity. Our findings of prolonged tail bleeding times in Cdc42^−/−^ mice (Fig. 8), consistent with observation by Pleines et al. [13], along with the observed impaired secretion and aggregation in Cdc42^−/−^ platelets (Figs. 5,6) clearly demonstrate that Cdc42 plays an essential role in regulation of platelet function and thus is critical for platelet mediated primary hemostasis.

In summary, our data implicate Cdc42 not only in reorganization of actin cytoskeleton including filopodia formation induced by fibrinogen, an integrin α_IIb_β_3_-dependent and CRP, a GPVI-dependent, pathway, but also in platelet aggregation. Regulation of granular secretion and Akt phosphorylation by Cdc42 appears to determine, at least in part, the extent of platelet aggregation induced by agonists that employ the GPVI and non-GPVI signaling pathways.

## References

[1] Etienne-Manneville S, Hall A (2002). Rho GTPases in cell biology.. Nature.

[2] Vidali L, Chen F, Cicchetti G, Ohta Y, Kwiatkowski DJ (2006). Rac1-null mouse embryonic fibroblasts are motile and respond to platelet-derived growth factor.. Mol Biol Cell.

[3] Erickson JW, Cerione RA (2001). Multiple roles for Cdc42 in cell regulation.. Curr Opin Cell Biol.

[4] Ridley AJ (2006). Rho GTPases and actin dynamics in membrane protrusions and vesicle trafficking.. Trends Cell Biol.

[5] Jones NP, Katan M (2007). Role of phospholipase Cgamma1 in cell spreading requires association with a beta-Pix/GIT1-containing complex, leading to activation of Cdc42 and Rac1.. Mol Cell Biol.

[6] Mulloy JC, Cancelas JA, Filippi MD, Kalfa TA, Guo F (2010). Rho GTPases in hematopoiesis and hemopathies.. Blood.

[7] Polakis PG, Snyderman R, Evans T (1989). Characterization of G25K, a GTP-binding protein containing a novel putative nucleotide binding domain.. Biochem Biophys Res Commun.

[8] Dash D, Aepfelbacher M, Siess W (1995). Integrin alpha IIb beta 3-mediated translocation of CDC42Hs to the cytoskeleton in stimulated human platelets.. J Biol Chem.

[9] Fox JE (1993). Regulation of platelet function by the cytoskeleton.. Adv Exp Med Biol.

[10] Azim AC, Barkalow K, Chou J, Hartwig JH (2000). Activation of the small GTPases, rac and cdc42, after ligation of the platelet PAR-1 receptor.. Blood.

[11] Vidal C, Geny B, Melle J, Jandrot-Perrus M, Fontenay-Roupie M (2002). Cdc42/Rac1-dependent activation of the p21-activated kinase (PAK) regulates human platelet lamellipodia spreading: implication of the cortical-actin binding protein cortactin.. Blood.

[12] Pula G, Poole AW (2008). Critical roles for the actin cytoskeleton and cdc42 in regulating platelet integrin alpha2beta1.. Platelets.

[13] Pleines I, Eckly A, Elvers M, Hagedorn I, Eliautou S (2010). Multiple alterations of platelet functions dominated by increased secretion in mice lacking Cdc42 in platelets.. Blood.

[14] Yang L, Wang L, Geiger H, Cancelas JA, Mo J (2007). Rho GTPase Cdc42 coordinates hematopoietic stem cell quiescence and niche interaction in the bone marrow.. Proc Natl Acad Sci U S A.

[15] Yang L, Wang L, Zheng Y (2006). Gene targeting of Cdc42 and Cdc42GAP affirms the critical involvement of Cdc42 in filopodia induction, directed migration, and proliferation in primary mouse embryonic fibroblasts.. Mol Biol Cell.

[16] Guo F, Hildeman D, Tripathi P, Velu CS, Grimes HL (2010). Coordination of IL-7 receptor and T-cell receptor signaling by cell-division cycle 42 in T-cell homeostasis.. Proc Natl Acad Sci U S A.

[17] Lockyer S, Okuyama K, Begum S, Le S, Sun B (2006). GPVI-deficient mice lack collagen responses and are protected against experimentally induced pulmonary thromboembolism.. Thromb Res.

[18] Szczur K, Zheng Y, Filippi MD (2009). The small Rho GTPase Cdc42 regulates neutrophil polarity via CD11b integrin signaling.. Blood.

[19] Akbar H, Kim J, Funk K, Cancelas JA, Shang X (2007). Genetic and pharmacologic evidence that Rac1 GTPase is involved in regulation of platelet secretion and aggregation.. J Thromb Haemost.

[20] Huzoor A, Wang W, Kornhauser R, Volker C, Stock JB (1993). Protein prenylcysteine analog inhibits agonist-receptor-mediated signal transduction in human platelets.. Proc Natl Acad Sci U S A.

[21] Huzoor A, Winegar DA, Lapetina EG (1991). Carboxyl methylation of platelet rap1 proteins is stimulated by guanosine 5′-(3-O-thio)triphosphate.. J Biol Chem.

[22] Quinton TM, Murugappan S, Kim S, Jin J, Kunapuli SP (2004). Different G protein-coupled signaling pathways are involved in alpha granule release from human platelets.. J Thromb Haemost.

[23] Cambien B, Bergmeier W, Saffaripour S, Mitchell HA, Wagner DD (2003). Antithrombotic activity of TNF-alpha.. J Clin Invest.

[24] Jaffer ZM, Chernoff J (2002). p21-activated kinases: three more join the Pak.. Int J Biochem Cell Biol.

[25] Smith SD, Jaffer ZM, Chernoff J, Ridley AJ (2008). PAK1-mediated activation of ERK1/2 regulates lamellipodial dynamics.. J Cell Sci.

[26] Hall A (1998). Rho GTPases and the actin cytoskeleton.. Science.

[27] Yang FC, Atkinson SJ, Gu Y, Borneo JB, Roberts AW (2001). Rac and Cdc42 GTPases control hematopoietic stem cell shape, adhesion, migration, and mobilization.. Proc Natl Acad Sci U S A.

[28] Wang Z, Oh E, Thurmond DC (2007). Glucose-stimulated Cdc42 signaling is essential for the second phase of insulin secretion.. J Biol Chem.

[29] Wang Z, Thurmond DC (2009). Mechanisms of biphasic insulin-granule exocytosis - roles of the cytoskeleton, small GTPases and SNARE proteins.. J Cell Sci.

[30] Momboisse F, Lonchamp E, Calco V, Ceridono M, Vitale N (2009). betaPIX-activated Rac1 stimulates the activation of phospholipase D, which is associated with exocytosis in neuroendocrine cells.. J Cell Sci.

[31] Li J, Luo R, Kowluru A, Li G (2004). Novel regulation by Rac1 of glucose- and forskolin-induced insulin secretion in INS-1 beta-cells.. Am J Physiol Endocrinol Metab.

[32] Hong-Geller E, Cerione RA (2000). Cdc42 and Rac stimulate exocytosis of secretory granules by activating the IP(3)/calcium pathway in RBL-2H3 mast cells.. J Cell Biol.

[33] Hong-Geller E, Holowka D, Siraganian RP, Baird B, Cerione RA (2001). Activated Cdc42/Rac reconstitutes Fcepsilon RI-mediated Ca2+ mobilization and degranulation in mutant RBL mast cells.. Proc Natl Acad Sci U S A.

[34] Thurmond DC (2009). Insulin-regulated glucagon-like peptide-1 release from L cells: actin' out.. Endocrinology.

[35] Dorsam RT, Kunapuli SP (2004). Central role of the P2Y12 receptor in platelet activation.. J Clin Invest.

[36] Cho MJ, Pestina TI, Steward SA, Lowell CA, Jackson CW (2002). Role of the Src family kinase Lyn in TxA2 production, adenosine diphosphate secretion, Akt phosphorylation, and irreversible aggregation in platelets stimulated with gamma-thrombin.. Blood.

[37] Kroner C, Eybrechts K, Akkerman JW (2000). Dual regulation of platelet protein kinase B.. J Biol Chem.

[38] Woulfe D, Jiang H, Morgans A, Monks R, Birnbaum M (2004). Defects in secretion, aggregation, and thrombus formation in platelets from mice lacking Akt2.. J Clin Invest.

[39] Kim S, Jin J, Kunapuli SP (2004). Akt activation in platelets depends on Gi signaling pathways.. J Biol Chem.

[40] Weng Z, Li D, Zhang L, Chen J, Ruan C (2010). PTEN regulates collagen-induced platelet activation.. Blood.

[41] Xiang B, Zhang G, Liu J, Morris AJ, Smyth SS (2010). A G(i) -independent mechanism mediating Akt phosphorylation in platelets.. J Thromb Haemost.

[42] Li Z, Zhang G, Le Breton GC, Gao X, Malik AB (2003). Two waves of platelet secretion induced by thromboxane A2 receptor and a critical role for phosphoinositide 3-kinases.. J Biol Chem.

[43] Kim S, Mangin P, Dangelmaier C, Lillian R, Jackson SP (2009). Role of phosphoinositide 3-kinase beta in glycoprotein VI-mediated Akt activation in platelets.. J Biol Chem.

[44] Yang L, Wang L, Kalfa TA, Cancelas JA, Shang X (2007). Cdc42 critically regulates the balance between myelopoiesis and erythropoiesis.. Blood.

[45] Shcherbina A, Cooley J, Lutskiy MI, Benarafa C, Gilbert GE (2010). WASP plays a novel role in regulating platelet responses dependent on alphaIIbbeta3 integrin outside-in signalling.. Br J Haematol.

[46] Teo M, Manser E, Lim L (1995). Identification and molecular cloning of a p21cdc42/rac1-activated serine/threonine kinase that is rapidly activated by thrombin in platelets.. J Biol Chem.

[47] Chang JC, Chang HH, Lin CT, Lo SJ (2005). The integrin alpha6beta1 modulation of PI3K and Cdc42 activities induces dynamic filopodium formation in human platelets.. J Biomed Sci.

[48] Jirouskova M, Jaiswal JK, Coller BS (2007). Ligand density dramatically affects integrin alpha IIb beta 3-mediated platelet signaling and spreading.. Blood.

